# Identification of Genomic Regions and Sources for Wheat Blast Resistance through GWAS in Indian Wheat Genotypes

**DOI:** 10.3390/genes13040596

**Published:** 2022-03-27

**Authors:** Rahul M. Phuke, Xinyao He, Philomin Juliana, Muhammad R. Kabir, Krishna K. Roy, Felix Marza, Chandan Roy, Gyanendra P. Singh, Aakash Chawade, Arun K. Joshi, Pawan K. Singh

**Affiliations:** 1 ICAR-Indian Agriculture Research Institute, Regional Station, Indore 452001, India; rahulphuke18@gmail.com; 2 ICAR-Central Institute for Cotton Research, Nagpur 440010, India; 3 International Maize and Wheat Improvement Center (CIMMYT), Apdo. Postal 6-641, Mexico City 06600, Mexico; x.he@cgiar.org; 4 Borlaug Institute for South Asia (BISA)/CIMMYT-India, NASC Complex, DPS Marg, New Delhi 110012, India; p.juliana@cgiar.org (P.J.); a.k.joshi@cgiar.org (A.K.J.); 5 Bangladesh Wheat and Maize Research Institute (BWMRI), Nashipur, Dinajpur 5200, Bangladesh; rezaulw@yahoo.com (M.R.K.); rkrishnaroy666@gmail.com (K.K.R.); 6 Instituto Nacional de Innovación Agropecuaria y Forestal (INIAF), La Paz 3798, Bolivia; femarza@hotmail.com; 7 Department of Plant Breeding and Genetics, Bihar Agricultural University, Sabour 813210, India; chandan.roy43@gmail.com; 8 ICAR-Indian Institute of Wheat and Barley Research, Maharaja Agarsain Marg, P.O. Box 158, Karnal 132001, India; GP.Singh@icar.gov.in; 9 Department of Plant Breeding, Swedish University of Agricultural Sciences, 23053 Alnarp, Sweden; aakash.chawade@slu.se

**Keywords:** wheat blast, GWAS, 2NS translocation, resistance breeding

## Abstract

Wheat blast (WB) is a devastating fungal disease that has recently spread to Bangladesh and poses a threat to the wheat production in India, which is the second-largest wheat producing country in the world. In this study, 350 Indian wheat genotypes were evaluated for WB resistance in 12 field experiments in three different locations, namely Jashore in Bangladesh and Quirusillas and Okinawa in Bolivia. Single nucleotide polymorphisms (SNPs) across the genome were obtained using DArTseq^®^ technology, and 7554 filtered SNP markers were selected for a genome-wide association study (GWAS). All the three GWAS approaches used identified the 2NS translocation as the only major source of resistance, explaining up to 32% of the phenotypic variation. Additional marker-trait associations were located on chromosomes 2B, 3B, 4D, 5A and 7A, and the combined effect of three SNPs (2B_180938790, 7A_752501634 and 5A_618682953) showed better resistance, indicating their additive effects on WB resistance. Among the 298 bread wheat genotypes, 89 (29.9%) carried the 2NS translocation, the majority of which (60 genotypes) were CIMMYT introductions, and 29 were from India. The 2NS carriers with a grand mean WB index of 6.6 showed higher blast resistance compared to the non-2NS genotypes with a mean index of 46.5. Of the 52 durum wheats, only one genotype, HI 8819, had the 2NS translocation and was the most resistant, with a grand mean WB index of 0.93. Our study suggests that the 2NS translocation is the only major resistance source in the Indian wheat panel analysed and emphasizes the urgent need to identify novel non-2NS resistance sources and genomic regions.

## 1. Introduction

Wheat is the most important cereal crop in the world, with a total acreage of 217 million hectares globally, and acts as a major source of nutrition and caloric intake [1]. According to an estimate by the Food and Agriculture Organization (FAO), the world will require an extra 100 million tons of wheat (~840 million tons) by the year 2050, under the challenges of biotic and abiotic stresses due to climate change [2]. At present, global wheat production will need to increase up to 60% in order to meet increasing demand [3]. Among the biotic stresses, wheat production is mainly affected by several fungal pathogen species causing frequent disease outbreaks globally. One such example is wheat blast (WB) caused by the fungal pathogen *Magnaporthe oryzae* pathotype *Triticum* (abbreviated as MoT) (anamorph *Pyricularia oryzae* pathotype *Triticum*) [4,5], affecting the major wheat growing areas in South America and recently South Asia, with a potential to also spread to other wheat production regions.

WB was first reported in Brazil in 1985, and subsequently spread to neighbouring countries, including eastern Bolivia, eastern Paraguay, and northern Argentina [6]. Under favourable weather conditions, up to 100% yield losses may occur [7,8]. Due to frequent WB epidemics, the wheat cropping area in the Cerrado region of Brazil dropped by 95% from 1987 to 2016 [9]. In 2016, an explosive WB outbreak occurred in Bangladesh for the first time outside South America, leading to dramatic yield losses in eight districts of Bangladesh [10,11]. In 2018, WB spread to Africa, with its first occurrence reported in Zambia, marking its expansion in the African continent [12]. The intercontinental spread of WB aroused serious concerns for the international wheat trade [6,8,9]. The occurrence of WB in Bangladesh poses a significant threat to the neighbouring countries like India and Pakistan, where WB vulnerable areas amounting to 7 million ha have been identified, with an estimated potential annual loss of 0.87–1.17 million tons [13]. These vulnerable areas include the densely populated and intensively cultivated Indo-Gangetic plains of Eastern India [14], which is one of the major wheat producing areas of India. Wheat varieties grown in India and other South Asian countries not only lack complete or durable resistance against WB, but also the known resistance sources are limited, which together make the wheat production system highly vulnerable to WB [5,15].

Breeding for WB-resistant genotypes is considered a sustainable and practical approach to control the disease. However, at present limited genetic studies have been conducted, and most of them are limited to seedling resistance in greenhouse as compared to field resistance, where host-pathogen interactions follow the gene-for-gene model [16]. Among the known MoT-specific resistance genes, *Rmg 2* and *Rmg 3* are temperature sensitive and specific to seedling resistance [17], whereas *Rmg 7* is effective at both seedling and adult plant stages but becomes ineffective at high temperatures [18]. Other genes like *Rmg1*, *Rmg 4*, *Rmg 5* and *Rmg 6* are resistant against non-MoT strains of *M. oryzae* [19,20,21]. The gene *Rmg 8* in combination with *Rmg GR119* confers good resistance to MoT isolates from Brazil and Bangladesh at the heading stage primarily in controlled environments, implying their promising utilization in breeding programs [16,22,23,24]. Apart from the *Rmg* genes, QTL mapping and genome-wide association studies (GWAS) have also identified numerous QTLs and marker trait associations (MTAs) on various wheat chromosomes. Goddard et al. [25] identified QTLs for seedling WB resistance on chromosomes 4A, 5A, and 2B from the Brazilian variety BR 18-Terena, explaining 17.8 to 19.6% of phenotypic variation. He et al. [26] identified the major role of the 2NS translocation on field WB resistance and additional minor QTLs on six chromosomes (1AS, 2BL, 3AL, 4BS, 4DL and 7BS). A similar study on two more bi-parental populations have recently been reported by the same authors, reporting the major effects of 2NS translocation along with several minor QTLs [27]. A GWAS using 184 South Asian wheat genotypes identified the major and stable effects of 2NS translocation on field WB resistance together with a few MTAs on chromosomes 1BS, 2AS, 6BS and 7BL [27]. Another GWAS study using 187 South Asian wheat germplasm revealed 40 significant markers associated with WB resistance, of which 33 (82.5%) were in the 2NS chromosome segment and one each on seven chromosomes (3B, 3D, 4A, 5A, 5D, 6A and 6B) [28]. The major effect of 2NS translocation region on field WB resistance was also reported by Juliana et al. [29] through GWAS analysis using 1106 lines from CIMMYT breeding nurseries, with additional MTAs identified on chromosomes 3BL, 4AL and 7BL. Likewise, Wu et al. [30] again confirmed the major role of 2NS translocation where 58 significant SNPs clustered in a 28.9-Mb interval, explaining phenotypic variation from 9.4 to 28.5%. Studies also reported host genes that recognize specific pathogen genes and provide resistance against WB. *Rwt3* and *Rwt4* are such non-host resistant genes in wheat that recognize pathogen genes *PWT3* and *PWT4*, respectively, resulting in an incompatibility (resistant) reaction [31,32]. Similarly, a hypothesis involving such interaction was proposed for the origin of WB in Brazil, which was due to large-scale cultivation of a variety lacking *Rwt3* that led to the susceptible reaction of majority wheat varieties to a mutated *Lolium-*like pathotype of *M. oryzae*, later known as MoT [33]. Even though 2NS translocation has consistently shown major effects on WB resistance, dependence on this single source of resistance is not suggested considering the breakdown of the 2NS-based resistance, which has already been witnessed in South America [5]. Hence the identification of novel sources of resistance is imperative. For a country like India, which neighbours Bangladesh where WB is expanding despite unfavourable weather conditions [4] and the disease being both air- and seed-borne, WB could easily move to India. Therefore, it is vital to screen Indian advanced breeding lines along with popular released cultivars for WB resistance to promote their cultivation and use in breeding programs as resistant donors. Hence the objectives of this study include (1) phenotyping Indian advanced breeding lines and released cultivars for adult plant WB resistance, and (2) identifying significant MTAs for WB resistance using GWAS. 

## 2. Materials and Methods

### 2.1. Plant Material and Phenotyping for Wheat Blast

A panel of 350 wheat genotypes was used in this study, which includes 298 bread wheat and 52 durum wheat accessions selected from released varieties and advanced breeding lines developed mainly during the last five years from 25 research centres throughout India. In addition to this, two checks, BARI Gom 33 (WB resistant) and BARI Gom 26 (WB susceptible), were included in the experiments in Bangladesh, and another set of checks, Urubo (WB resistant) and Atlax (WB susceptible), was used in Bolivia. The genotypes were evaluated for field resistance to WB in three different locations including Quirusillas and Okinawa in the department of Santa Cruz, Bolivia, and Jashore in Bangladesh. Quirusillas is located at high altitude of 1496 m above sea level (masl) with wheat cropping cycles from December to April, Okinawa is at a low altitude of 267 masl with cropping cycles ranging from May to August and Jashore is located in the southwestern region of Bangladesh at an altitude of 7 masl, with cropping cycles from December to April. The trials were performed in the 2019–2020 and 2020–2021 cropping seasons in Quirusillas, the 2020 cropping season in Okinawa and the 2018–2019, 2019–2020 and 2020–2021 cropping seasons in Jashore. The GWAS panel was evaluated in two sowing dates in each season approximately 14 days apart, resulting in 12 experiments in total, which were named based on location, cropping cycle/year and sowing dates, where “Oki” stands for Okinawa, “Quir” stands for Quirusillas, “Jash” for Jashore, “19” for the 2018–2019 cycle, “20” for the 2019–2020 or 2020 cycle, “21” for the 2020–2021 cycle, and “a” and “b” for the first and second sowing, respectively. For example, “Jash19a” represents the first sown experiment in 2018–2019 at the Jashore location. 

Each genotype was sown in 1-m long double rows, each with a 20-cm row-to-row spacing. The inoculum used was a mixture of local MoT isolates with high aggressiveness including OKI1503, OKI1704, QUI1505, QUI1601, QUI1612 in Bolivia and BHO17001, MEH17003, GOP17001.2, RAJ17001, CHU16001.3, JES16001 in Bangladesh. These isolates were grown on oatmeal agar medium following the protocol by He et al. [26]. The inoculum was applied at the concentration of 80,000 spores mL^−1^ at anthesis and two days after anthesis, with a backpack sprayer. A misting system was equipped to keep a humid microenvironment which is favourable for WB development, working from 8 a.m. to 7 p.m. in Bolivia and 9 a.m. to 5 p.m. in Bangladesh, with 10 min of spraying hourly. WB evaluations were performed at 14 or 21 days after the first inoculation, when the total and infected spikelets on 10 spikes tagged at anthesis were recorded. The WB index was calculated with the formula WB index = incidence × severity. In addition to the WB index, days to heading (DH) and plant height (PH) were recorded in all the experiments. The mean WB index was calculated using data across all the environments. ANOVA was performed with the PROC GLM module in SAS ver 9.2, and heritability estimates were calculated using the formula $H^{2}=\sigma^{2}g/(\sigma^{2}g+\sigma^{2}g*y/y+\sigma^{2}g*s/s+\sigma^{2}e/sy)$ for experiments in Quirusillas and Jashore and $H^{2}=\sigma^{2}g/(\sigma^{2}g+\sigma^{2}e/s)$ for experiments in Okinawa, where $\sigma_{g}^{2}\;$ represents genetic variance, $\sigma_{g*y}^{2}\;$ genotype-by-year interaction, $\sigma_{g*s}^{2}$ genotype-by-sowing interaction, $\sigma_{e}^{2}$ error variance, *y* the number of years, and *s* the number of sowings. We also obtained Pearson correlation coefficients for WB indices among the 12 experiments, and those between WB indices and phenological traits DH and PH, using GenStat software, 17th edition (VSN, International, Hemel Hempstead, UK). The phenotypic-data-based principal component analysis (PCA) was calculated with PAST software ver. 3.01 [34].

### 2.2. Genotyping

The panel of 350 wheat accessions was genotyped with the DArTseq^®^ technology at the Genetic Analysis Service for Agriculture (SAGA) at CIMMYT, Mexico, and DNA extraction and DArT sequencing was done as per the protocol used in Li et al. [35]. Markers with minor allele frequency less than 10% (2804 markers) and more than 30% missing datapoints (96 markers) were excluded from further analysis. The SNP markers were named by chromosome location and their physical location in the Chinese Spring reference genome Refseq v1.0, e.g., 2B_180938790 indicates a marker on chromosome 2B at 180938790 bp. Four STS markers in the 2NS translocation region were also used to evaluate their association with WB resistance and suitability for marker-assisted selection (MAS), including *Ventriup-LN2* reported by Helguera et al. [36], *WGGB156* and *WGGB159* by Wang et al. [24] and *cslVrgal3* from a follow-up study of Seah et al. [37] (E. Lagudah, pers. comm.).

### 2.3. Linkage Disequilibrium, Kinship and Population Structure Analysis

For the 298 bread wheat genotypes in the panel, linkage disequilibrium (LD) parameters R^2^ among the SNP markers were calculated using TASSEL 5 (http://www.maizegenetics.net, accessed on 25 March 2022), and the LD estimates as the allele frequency correlation (R^2^) among SNP markers were plotted against the physical distances in mega base pairs (Mb) across the chromosomes. A kinship matrix and clusters among individual genotypes were calculated using all SNP markers, and a heat map was generated using the classical equation from Van Randen (2008) in the program R. For population structure analysis, the numeric transformation of genotypic data was performed using XLSTAT (2010) as per the required format of the Structure 2.3.4 software [38]. The admixture model was adjusted with a burn-in period length of 100,000 followed by 500,000 marker chain Monte Carlo (MCMC) replications. The subpopulation test range was kept from K1 to K5, each with five interactions (runs). The ΔK approach was used to access the actual subpopulations (Earl, 2012). ΔK was confirmed by the method detailed in [39] using the STRUCTUREHARVERSTER program [40]. The average logarithm of the probability of the observed likelihood [LnP(D)] was calculated along with the standard deviation from the output summary. LnP(D) for each step of the MCMC was calculated for each class (K = 1 to 5) by computing the log likelihood for the data.

### 2.4. GWAS Analysis

GWAS was conducted for the 298 bread wheat genotypes using 7554 SNP markers using an MLM (mixed linear model) in TASSEL 5 [41]. The Q + K model that considers both Kinship (K matrix) and population structure was adopted. In addition, the multilocus mixed model (MLMM) and fixed and random model circulating probability unification (FarmCPU) model were also analysed using the R software package GAPIT v 3.5 [42]. The *p*-value, additive effect and percentage variation explained by each marker were obtained and Manhattan plots with the −log10 *p*-values of the markers were plotted using the ‘R’ package CMplot [43]. GWAS was conducted individually for the 12 experiments and the pooled data, and the markers were declared to be significant using Bonferroni correction with significant cut-off at an α level of 0.20 (*p*-values 2.64 × 10^−5^); additionally, putative MTAs were reported at an increased *p* value of 0.001. 

## 3. Results

### 3.1. Field Phenotyping for Wheat Blast

**Figure 1:**
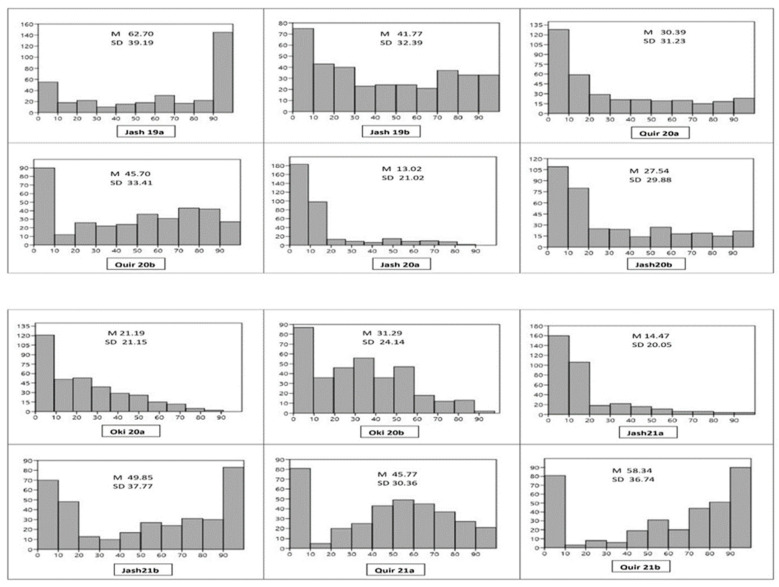
Histogram of wheat blast index (X axis) and number of genotypes (Y axis) in 12 individual experiments. M (Grand mean), SD (Standard deviation), Jash (Jashore), Quir (Quirusillas), Oki (Okinawa), 19 (2018–2019 cycle), 20 (2019–2020 or 2020 cycle), 21 (2020–2021), a (first sowing), b (second sowing).

Among the 12 experiments, the highest mean WB index was observed in the Jash19a experiment (62.70) followed by Quir21b (58.34), and the lowest in Jash20a (13.02). Almost all experiments showed the bimodal distribution of genotypes except Jash20a and Jash21a, where the WB indices were low (Figure 1). In all the experiments, the second sowing showed higher WB infection compared to the first, except for Jash19a and Jash19b, where a reverse trend was observed. The distribution of WB indices indicated that Jash21a has the most genotypes (50%) with a blast index of 0, while Jash19a and Jash19b had the least number of such genotypes (13.9%). Overall, across all the experiments, 21.3% of genotypes had an average WB index less than 10 and were classified as highly resistant (Supplementary Table S1a,b). ANOVA indicated that variance due to the genotypes ($\sigma_{g}^{2}$) was highly significant in all three locations, besides the years ($\sigma_{y}^{2}$) in Jashore and Quirusillas, and sowing ($\sigma_{s}^{2})\;$ in Jashore and Okinawa. The genotype × year interaction ($\sigma_{g*y}^{2}$) was significant in Jashore and non-significant in Quirusillas, while genotype × sowing interaction ($\sigma_{g*s}^{2}$) was non-significant in both Jashore and Quirusillas (Table 1). As per broad-sense heritability [44], WB resistance was highly heritable in Jashore (0.78) and Quirusillas (0.72) but was moderately heritable in Okinawa (0.52). 

**Figure 2:**
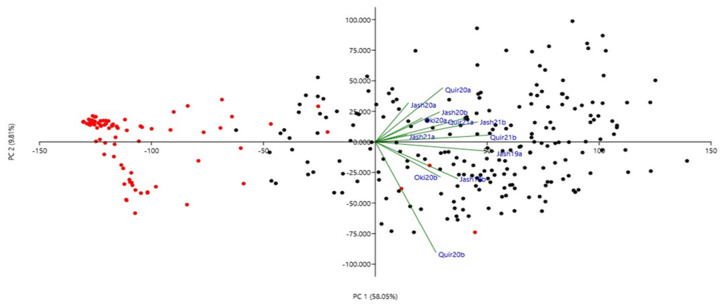
Principal component analysis (PCA) of 298 Indian bread wheat genotypes using wheat blast index across 12 experiments. Red and black symbols denote 2NS and non-2NS genotypes, respectively. Refer to Supplementary Figure S1 for decoding genotype labels.

The phenotypic correlations for WB indices among the 12 experiments were all significant at *p* ≤ 0.01 (Table 2) except among the Jash20a and Quir20b. The highest correlation (r = 0.78) was observed between Quir21a and Quir21b and the lowest (r = 0.08) was between Jash20a and Quir20b. Among all the experiments, Jash20a and Jash21a with low disease pressure showed lower correlations with other experiments, while others such as Jash19a, Jash19b, Quir20b, Quir21a and Quir21b exhibited better correlations among one another. However, based on PCA analysis at the first two PCs, Quir20b showed the least association with the remaining experiments (Figure 2). The first PC indicated the presence of two major groups, a small group with 2NS carriers and a major group carrying mostly non-2NS genotypes (Figure 2). As for the phenological traits, a significant negative correlation was observed between WB and days to heading (DH) in five experiments (Jash19a, Jash19b, Quir20b, Oki20b and Jash21b) with r values ranging from −0.17 to −0.28 and significant positive correlation in Jash20a (r = 0.27), whereas nonsignificant correlation was observed in other six experiments (Quir20a, Jash20b, Oki20a, Jash21a, Quir21a and Quir21b). As for plant height (PH), significant negative correlation was observed with WB in three experiments (Quir20, Oki20b and Jash21a) with moderate r value around −0.40, whereas nonsignificant correlations were found in other experiments (Supplementary Table S2). 

All correlations are significant at *p* ≤ 0.01, except for that between Jash20a and Quir20b

### 3.2. SNP Distribution, Population Structure and Linkage Disequilibrium Analysis

**Figure 3:**
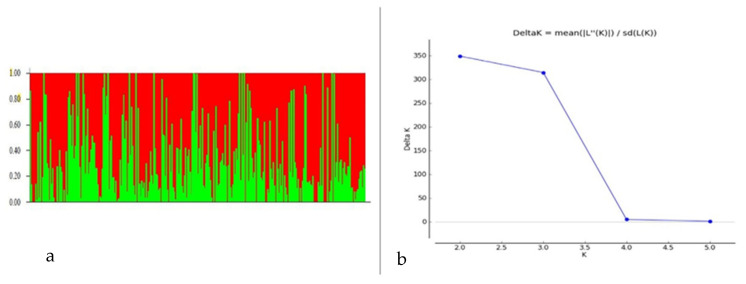
Population structure analysis of the 298 Indian bread wheat genotypes. (**a**) Bar plot indicating membership coefficient (Q value at Y axis). (**b**) Estimation of hypothetical sub-populations using ΔK-values; the maximum value of delta K occurred at K = 2.

Among 7554 SNP markers selected for GWAS in the 298 bread wheat genotypes, 31.25% were from the A genome, 37.86% from the B genome, 11.04% from the D genome and 19.20% from unknown chromosomes. The highest number of markers were located on chromosome 2B (557 markers) followed by 7B (504 markers), and the lowest were on chromosome 4D (45 markers). Population structure analysis based on the K mean cluster approach divided the bread wheat population into two major groups, designated as WBpop-1 and WBpop-2 (Supplementary Table S1 and Figure 3). The WBpop-1 accommodated 73 genotypes, of which 40 were CIMMYT-derived and 33 were from Indian breeding programs. WBpop-2 had 225 genotypes, including 105 CIMMYT-derived and 120 Indian genotypes. WBpop-1 had 17.8% 2NS genotypes compared to 33.2% in WBpop-2. The grouping of genotypes was mostly based on pedigree, e.g., the most frequent parent for WBpop-1 accessions was “Sokoll”, while for WBpop-2 were “Kauz”, “Kachu” and “Milan”. It is noteworthy that the parents PBW550, Francolin, Trap and Brambling were exclusively observed in the pedigree of WBpop-2 accessions. Genotypes with identical or close pedigrees showed maximum similarity and formed compact clusters, such as HD3321 vs. PBW804 in WBpop-1 and HD3232 vs. K1315 in WBpop-2. Kinship analysis (Supplementary Figure S2) also indicated two main groups; one small group carrying 23 genotypes, most of them having 2NS translocation, and one large group of 275 genotypes with and without 2NS translocation. The large group was further divided into seven subgroups, each having accessions with common ancestries. The average extent of LD, considered as physical distance taken for the decay of R^2^ to reach a critical value of 0.10 across the genome, was approximately 10 Mb (Supplementary Figure S3).

### 3.3. GWAS for Blast Resistance in the 298 Bread Wheat Genotypes

**Figure 4:**
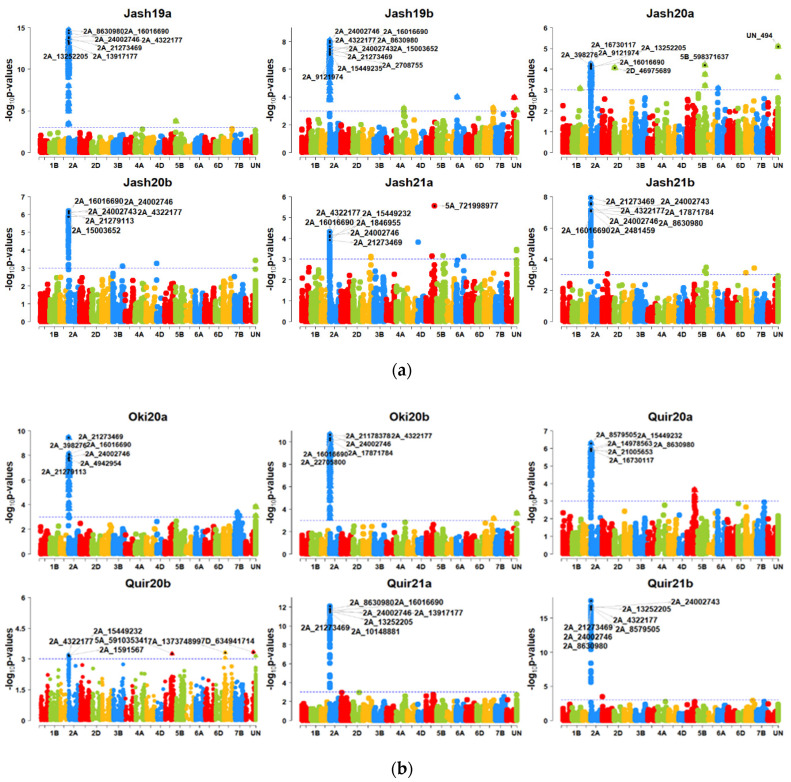
(**a**) Manhattan plots for wheat blast indicating the marker-blast associations obtained using genome-wide association mapping for six experiments in Jashore. Jash (Jashore), 19 (2018–2019 cycle), 20 (2019–2020 cycle), 21 (2020–2021 cycle), a (first sowing), b (second sowing). (**b**) Manhattan plots for wheat blast indicating the marker-blast associations obtained using genome-wide association mapping for two experiments in Okinawa and four experiments in Quirusillas. Quir (Quirusillas), Oki (Okinawa), 20 (2019–2020 cycle or 2020), 21 (2020–2021 cycle), a (first sowing), b (second sowing).

With the MLM model at *p* = 2.64 × 10^−5^ (Bonferroni cut-off), a large number of SNP markers were significant on the 2NS translocation region, including most significant markers like 2A_21273469, 2A_4322177, 2A_16016690, 2A_24002746 and 2A_13917177, while only SNP UN_494 (unknown chromosome) and SNP 5A_721998977 (5AL chromosome) were not in the 2NS translocation (Figure 4a,b). However, this model was ineffective in identifying significant MTAs in Quir20b, and it could identify only one significant MTA each for Jash20a and Jash21a that was not located in the 2NS translocation. However, at *p* = 0.001, significant MTAs on 2NS were identified in all 12 individual experiments and several markers outside the 2NS translocation were also identified. However, none of them was repeatedly detected across the experiments (Supplementary Table S3). At *p* value 0.001, the highest number of significant MTAs were identified in experiment Jash21b (64 MTAs), followed by Jash19b and Oki20a (57 MTAs) and the lowest number of MTAs were identified in experiment Quir20b (10 MTAs). With the MLMM model, 14 MTAs were identified at the threshold *p* = 2.64 × 10^−5^, among which 12 MTAs were on the 2NS translocation while other two MTAs (5A_721998977 and UN_716) were located on chromosome 5AL and an unknown chromosome, respectively. When the *p*-value cut-off was 0.001, 112 significant MTAs were detected in total, from which 15 MTAs were from the 2NS translocation and those remaining were from other chromosomes. It is noteworthy that one MTA 7A_750227572 on chromosome 7AL was repeatedly detected in two individual experiments and in the pooled dataset. The GWAS analysis with the FarmCPU model could identify 62 significant MTAs in the 12 individual experiments at a *p*-value cut-off of 2.64 × 10^−5^, which were located on the 2NS translocation and 15 other chromosomes. Of the non-2NS MTAs, six were repeatedly identified in two or more experiments (Table 3). In addition, the combination of favourable alleles for three SNPs, namely 2B_180938790 on chromosome 2BS, 7A_752501634 on 7AL and 5A_618682953 on 5AL showed better resistance, as 14 bread wheat genotypes (Table 4) in total carrying such alleles showed a WB index of less than 30%.

GWAS was also separately conducted in the panel with the 209 non-2NS genotypes. The MLM model identified 64 MTAs at a *p* value cut-off of 0.001 on chromosomes 1B, 2B, 2D, 3A, 3B, 4A, 4B, 4D, 5A, 5B, 6A, 6B, 6D, 7A, 7B and an unknown chromosome (Supplementary Table S4). The maximum number of MTAs were located on chromosome 5A (15 MTAs), followed by those on unknown chromosomes (14 MTAs) and chromosome 7A (7 MTAs). Among these MTAs, only four on 5AL were repeatedly detected in two or more experiments, and two of them, 5A_721998977 and 5A_618682953, were significant at a *p* value cut-off of 2.64 × 10^−5^. 

### 3.4. 2NS Translocation and Wheat Blast Resistance in Wheat

**Figure 5:**
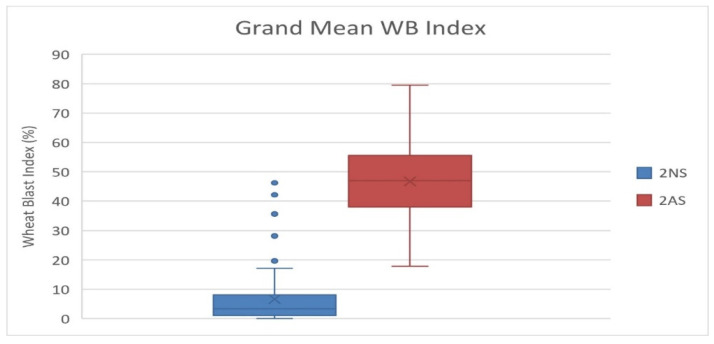
Boxplot for phenotypic variation in the 2NS vs. 2AS genotypes based on grand mean WB data over 12 experiments.

Among the studied 298 bread wheat genotypes, 89 (29.9%) genotypes showed the presence of 2NS translocation and majority of them (60 genotypes) were CIMMYT introductions, and 29 were from India (Table S1). These genotypes include several released cultivars in India, i.e., DBW 88, DBW 168, DBW 173, DBW 187, DBW 222, DBW 252, DBW 303, DPW621/50, HD 2967, HD 3043, HD 3059, HD 3171, HD 3249, HD 3293, HI 1605, HI 1620, MACS 6478, PBW 752, WH1105 and WH 1270. A grand mean WB index of 6.6 was recorded for the 2NS genotypes, in comparison to 46.5 for the 2AS genotypes (Figure 5). In the case of 2NS genotypes, we observed that eight accessions had a grand mean WB index of zero, 66 were between zero and 10, and 12 between 10 and 30. However, three 2NS genotypes showed a higher WB index of 38.50 (DBW 283), 42.13 (MP3516) and 46.23 (NW 7049). In the case of 2AS genotypes, we observed that no genotype showed resistance based on an arbitrary threshold of 15%. The most resistant 2AS genotype was MACS 6736 having a mean WB index of 17.79. Most likely its resistance was due to desirable alleles at three significant SNPs, i.e., 2B_180938790 on chromosome 2BL, 5A_618682953 on chromosome 5AL and 7A_752501634 on chromosome 7AL. Among the 52 durum wheat genotypes, genotype HI 8819 carrying 2NS was observed as the most resistant with a grand mean WB index of 0.93. This genotype showed zero infection in 11 out of the 12 experiments, whereas no genotype among the remaining 51 (all non-2NS genotypes) showed resistance based on an arbitrary threshold of 15%. The pedigree details of HI 8819 (Supplementary Figure S4) reveal it has combination of diverse rust resistance lines, including Altar 84 and Flamingo “s” form CIMMYT breeding program, Bijaga Yellow and Bijaga Red form Indian breeding program, a Canadian durum wheat cultivar Hercules having resistance to races of leaf and stem rust and loose smut, and the popular middle eastern durum line Gaza carrying the adult plant resistant (APR) gene for leaf rust. However, it is not confirmed which parent is contributing to the WB resistance in HI 8819 as they are yet to be screened for WB reaction.

## 4. Discussion

The recent outbreak of WB in Asia is a threat to global food security [45]. WB can easily spread through infected seed and airborne spores indicating high chances of introduction in India through the 4096 km international border between India and Bangladesh. The most sustainable and effective way to tackle this issue is through identification and development of wheat blast resistant cultivars. The 350 genotypes evaluated for their resistance to WB showed significant differences in all three locations, indicating an ample variation for resistance. The phenotypic correlation for WB indices among the 12 experiments varied from low to high, and generally high correlation values were observed among experiments with sufficient WB disease pressure. The good correlation between experiments in Bangladesh and Bolivia such as between Jash19a and Quir21b (r = 0.68, *p* ≤ 0.01) and between Jash19a and Oki20b (r = 0.63, *p* ≤ 0.01) indicates similar WB disease pressure in the two countries, just as earlier reported by Juliana et al. [29]. Both the highest and the lowest mean WB indexes were observed in the Jashore location of Bangladesh, indicating the role of environment in WB infection. Whenever there is rainy and warm weather conditions during the heading stage of a wheat crop, the occurrence and development of WB is enhanced and vice versa [46]. It is often recommended that a population used for genetic studies on disease resistance should have low variation in phenological traits like DH and PH, knowing their influence on disease infection in field conditions, as reported earlier for Fusarium head blight [47] and spot blotch [48]. However, this was not observed in the present study on WB resistance, owing to the low variation in the panel in the two traits, as earlier reported by He et al. [27] in different panels of CIMMYT and South Asian wheat genotypes.

Multi-environment GWAS analysis in the 298 bread wheat genotypes provided valuable insight into the genomic regions associated with WB resistance in Indian wheat genotypes. Many markers showing significant association with WB resistance in multiple environments were in the 2NS translocation, exhibiting strong phenotypic effects on WB resistance. These results align with previous reports documenting wheat lines with the 2NS chromosomal segment from *Aegilops ventricose* [7], and the large difference in WB indices between 2NS and non-2NS genotypes [26,29]. Among the 2NS carriers, the frequency of CIMMYT lines (76%) was much higher than that of Indian lines (24%), which agrees with the fact that the 2NS translocation has been widely utilized in the CIMMYT breeding program over past years [29]. The CIMMYT 2NS genotypes often have parents like MILAN, KAUZ and KACHU in their pedigree. MILAN is a well-known donor for WB resistance and has been widely used for the development of WB-resistant cultivars in South America [49]. Milan was the original source of 2NS translocation in the CIMMYT breeding program and was used in the development of Kachu (KAUZ//ALTAR 84/AOS/3/MILAN/KAUZ/4/VEE/KOEL), which was also widely used as a parent in the CIMMYT breeding program [29]. It is worth noting that the WB index of the 2NS genotypes varied from 0 to 46.23, indicating the background-dependence nature of 2NS translocation, which is in consistent with the reports of Cruz et al. [7]. In the case of 2NS linked STS markers used in this study, all four STS markers were significant at a *p* value of 2.64 × 10^−5^; however, we observed that the STS markers *Ventriup-LN2* and *cslVrgal3* were more efficient in diagnosing 2NS compared to WGGB156 and WGGB159 in the 298 bread wheat, as well as in 52 durum genotypes. A similar observation in Indian germplasm was earlier reported by He et al. [27]. It is remarkable that among the resistant genotypes identified in this study, several accessions like DBW 187, DBW 252, HD 3171 and HD 3293 were recently released in the north-eastern plain zone of India (NEPZ) [50,51,52], which can be recommended for cultivation in the state of West Bengal, which borders Bangladesh and has high risk of WB epidemics. In the case of durum wheat, HI 8819 was the only 2NS-carrying genotype that showed excellent WB resistance, which could be used as a 2NS donor in durum breeding programs in India. 

Besides 2NS translocation, we identified six markers that were significant in more than one experiment. They are located on chromosomes 2BS, 3BL, 4DS, 5AL, 6AS and 7AL, but had much lower phenotypic effects in comparison to 2NS translocation. However, the non-2NS genotypes having a combination of favourable alleles for MTAs on 2BS (2B_180938790), 5AL (5A_618682953) and 7AL (7A_752501634) and showing a low WB index clearly indicates the additive effects of these alleles on WB resistance, similar to that reported by He et al. [26,53]. Of the three SNPs, only 5A_618682953 at 618.6 Mb on 5AL is close to previously reported SNPs by Roy et al. [28] at 582.8 Mb and by Juliana et al. [29] at 665.8 Mb, whereas the other two on 2BS and 7AL appear to be new. To investigate minor MTAs that might have been masked by the 2NS translocation, a GWAS was conducted only for the 2AS genotypes, which led to multiple MTAs outside the 2NS region, especially on chromosome 5AL. These MTAs could be deployed in the breeding program once validated in other studies, to alleviate the strong selection pressure on 2NS, considering the emergence of 2NS-virulent isolates in South America [6,54].

From this study it is obvious that 2NS is the only major source of WB resistance in Indian wheat germplasm, and a few 2NS genotypes have already been released for cultivation in the north-eastern plain zone of India. However, since depending on only one resistance source with strong phenotypic effect may lead to the breakdown of WB resistance [6,55], more attention should be given to the identification and utilization of non-2NS loci. The strategy of using minor genes after the breakdown of major genes was successfully used in wheat rust [56]. In this regard, the non-2NS MTAs identified on chromosomes 7AL (7A_781518015, 7A_752501634 and 7A_750227572) and 5AL (5A_618682953 and 5A_721998977) need to be further validated for their potential use in breeding. So far, genetic studies on WB resistance have been mostly limited to elite germplasm, hence there is a need to investigate landraces, synthetic wheat and wild relatives for novel sources/genes for WB resistance. A good example is the landrace GR119 [23] which is a novel resistance source against WB and carries two genes *Rmg 8* and *Rmg GR119* for WB resistance. Identifying novel MTAs using non-2NS genotypes is suggested for future GWAS studies to avoid their effects being masked by 2NS translocation. Since non-MoT *M. oryzae* pathotypes *Oryza* for rice blast and *Lolium* for grey leaf spot in ryegrass have shown a potential threat to wheat [57], non-host resistance genes against these pathotypes must also be surveyed and utilized if they are of low frequencies. This is especially relevant to India, where rice, which suffers greatly from blast disease, is grown in rotation with wheat over a huge area.

## 5. Conclusions

The present study concludes that in the Indian wheat germplasms screened, 2NS is the only major source of resistance to WB. The minor MTAs identified outside 2NS translocation showing additive effects could be used together through MAS considering their low phenotypic effects. The WB-resistant genotypes carrying 2NS translocation should be recommended for cultivation for the effective management of the WB disease. Furthermore, there is an urgent need for the identification of non-2NS resistant sources with major phenotypic effects. 

## Figures and Tables

**Table 1:** Analysis of variance for wheat blast index in three locations and the corresponding heritability estimates.

Location	Source	DF	Mean Squares	F	*p*	Heritability
Jashore	Genotype	349	3047.65	6.4	<0.0001	0.78
	Year	2	179,096.65	377.83	<0.0001	
	Genotype × Year	696	658.46	1.39	<0.0001	
	Genotype × Sowing	349	400.88	0.85	0.962	
	Sowing (Year)	2	140,829.27	297.1	<0.0001	
	Error	694	474.01			
Quirusillas	Genotype	349	2504.7084	4.86	<0.0001	0.72
	Year	1	58,706.8672	114.09	<0.0001	
	Genotype × Year	348	610.2701	1.18	0.0663	
	Genotype × Sowing	349	613.2954	1.18	0.0644	
	Sowing (Year)	1	73.5923	0.06	0.8146	
	Error	324	517.564			
Okinawa	Genotype	349	696.319	2.11	<0.0001	0.52
	Sowing	1	12,235.2133	34.51	<0.0001	
	Error	347	333.9891			

**Table 2:** Person correlation coefficients among 12 different environments for wheat blast index.

	Jash19a	Jash19b	Quir20a	Quir20b	Jash20a	Jash20b	Oki20a	Oki20b	Jash21a	Jash21b	Quir21a
Jash19b	0.65										
Quir20a	0.43	0.38									
Quir20b	0.40	0.35	0.20								
Jash20a	0.33	0.28	0.44	0.08							
Jash20b	0.47	0.41	0.37	0.21	0.44						
Oki20a	0.47	0.36	0.60	0.26	0.45	0.42					
Oki20b	0.63	0.60	0.31	0.48	0.16	0.41	0.36				
Jash21a	0.33	0.34	0.44	0.29	0.44	0.46	0.56	0.29			
Jash21b	0.57	0.51	0.53	0.37	0.46	0.50	0.57	0.51	0.54		
Quir21a	0.66	0.52	0.60	0.34	0.39	0.49	0.63	0.56	0.40	0.63	
Quir21b	0.68	0.50	0.56	0.41	0.32	0.45	0.57	0.61	0.37	0.61	0.78

**Table 3:** Markers significantly associated with wheat blast resistance through genome wide association mapping using mixed linear model (MLM), multi locus mixed model (MLMM) and fixed and random model circulating probability unification (FarmCPU).

Algorithm	Marker	Chromosome	Position (Mb)	*p* Value	*R* ^2^	Experiment
All	Multiple SNPs	2NS	195997—29397023	7.41 × 10^−53^ to 2.55 × 10^−5^	0.05 to 0.32	All
Farm CPU	2B_180938790	2BS	180938790	1.67 × 10^−6^ to 5.06 × 10^−6^	0.03 to 0.04	Jash20b, GM
Farm CPU	3B_794537258	3BL	794537258	8.89 × 10^−6^ to 1.67 × 10^−5^	0.005 to 0.03	Quir 20a, GM
Farm CPU	4D_25473616	4DS	25473616	1.10 × 10^−5^ to 2.22 × 10^−5^	0.04 to 0.05	Jash 21a, Oki 20a
Farm CPU	5A_618682953	5AL	618682953	5.84 × 10^−7^ to 1.06 × 10^−5^	0.05 to 0.06	Oki 20b, Quir 21b
Farm CPU	6A_75053670	6AS	75053670	3.11 × 10^−8^ to 5.07 × 10^−7^	0.04 to 0.05	Jash19a, Quir21a
Farm CPU	7A_752501634	7AL	752501634	5.41 × 10^−6^ to 2.17 × 10^−5^	0.004 to 0.02	Jash19b, Jash21b

Jash (Jashore), Quir (Quirusillas), Oki (Okinawa), 19 (2018–2019 cycle), 20 (2019–2020 or 2020 cycle), 21 (2020–2021 cycle), a (first sowing), b (second sowing), GM (grand mean).

**Table 4:** List of non-2NS genotypes showing moderate resistance to wheat blast.

Entry	Origin	Pedigree	WB Index (%)
MACS6736	Indian	NI 5439/HD2934	17.79
DBW297	CIMMYT	SOKOLL/3/PASTOR//HXL 7573/2*BAU/4/MASSIV/PPR47.89C	21.10
DBW286	Indian	DBW 43/DPW 621-50	22.71
DBW273	CIMMYT	FRANCOLIN #1*2//ND 643/2* WBLLI	22.78
KRL423	CIMMYT	SOKOLL/3/PASTOR//HXL7573/2*BAU/4/GLADIUS	23.71
PBW805	CIMMYT	OASIS/SKAUZ//4*BCN/3/2*PASTOR/4/PBW631	24.29
UP3028	CIMMYT	BECARD#1/CIRNO C 2008//BECARD	24.80
PBW804	CIMMYT	SOKOLL/3/PASTOR//HXL7573/2*BAU/4/HUW234 + LR34/PRINIA//PBW34 3*2/KUKUNA/3/ROLF07	25.45
PBW773	CIMMYT	FRANCOLIN#1*2/KIRITATI	27.98
HD3339	CIMMYT	FRANCOLIN#1//WBLL1*2/BRAMBLING	28.06
WH1259	CIMMYT	SNB//CMH79A.955/3*CNO79/3/ATTILA/4/CHEN/AE.SQUARROSA (TAUS)//BCN/3/2*KAUZ/5/KINGBIRD#1	28.73
MP1361	CIMMYT	CHEN/AEGILOPS SQUARROSA (TAUS)//BCN/3/BAV92/4/BERKUT/5/BAVIS/JWS140	29.68
JAUW672	CIMMYT	SERI.18*2/3/KAUZ*2/BOW//KAUZ/4/CROC	29.69
MP1360	CIMMYT	SOKOLL/3/PASTOR//HXL7573/2*BAU/4/GLADIUS/MP 1285	29.93

## Data Availability

The original genotypic data of this study can be found here: https://hdl.handle.net/11529/10548642, accessed on 25 March 2022.

## Supplementary Materials

The following supporting information can be downloaded at: https://www.mdpi.com/article/10.3390/genes13040596/s1, Supplementary Table S1a: Wheat blast data indices of 298 bread wheat genotypes for 12 individual experiments and pooled across 12 experiments (grand mean), Supplementary Table S1b: Wheat blast data indices of 52 durum wheat genotypes for 12 individual experiments and pooled across 12 experiments (grand mean), Supplementary Table S2: phenotypic correlation of phenological traits days to heading (DH) and plant height (PH) with WB index in individual experiments, Supplementary Table S3: Markers significantly associated with wheat blast resistance using MLM, MLMM and FarmCPU models at *p* ≤ 0.001 across 12 individual experiments and pooled across experiments (grand mean), Supplementary Table S4: Markers significantly associated with wheat blast resistance in 219 non-2NS genotypes using MLM model at *p* ≤ 0.001, Supplementary Figure S1: Principal component analysis of 298 bread wheat lines with genotypes labels for blast index in all the environments. Genotypes with red symbol are 2NS-positive, Supplementary Figure S2: Heatmap and dendrogram of kinship matrix based on 7554 SNP markers and 298 bread wheat genotypes, Supplementary Figure S3: Scatter plot showing linkage disequilibrium (LD) decay in the 298 bread wheat genotypes. Physical distance in Mb is plotted against the LD estimate (R2) for pairs of markers, Supplementary Figure S4: Pedigree profile of durum wheat genotype HI 8819.
