# Supplementary material for: Identification of Genomic Regions and Sources for Wheat Blast Resistance through GWAS in Indian Wheat Genotypes
Source: Genes (Basel). 2022 Mar 27;13(4):596. doi: 10.3390/genes13040596 (PMC9025667; doi:10.3390/genes13040596)
Supplement: Supplementary file 1 [file genes-13-00596-s001.zip › Figure S2. Kinship Matrix.pdf]

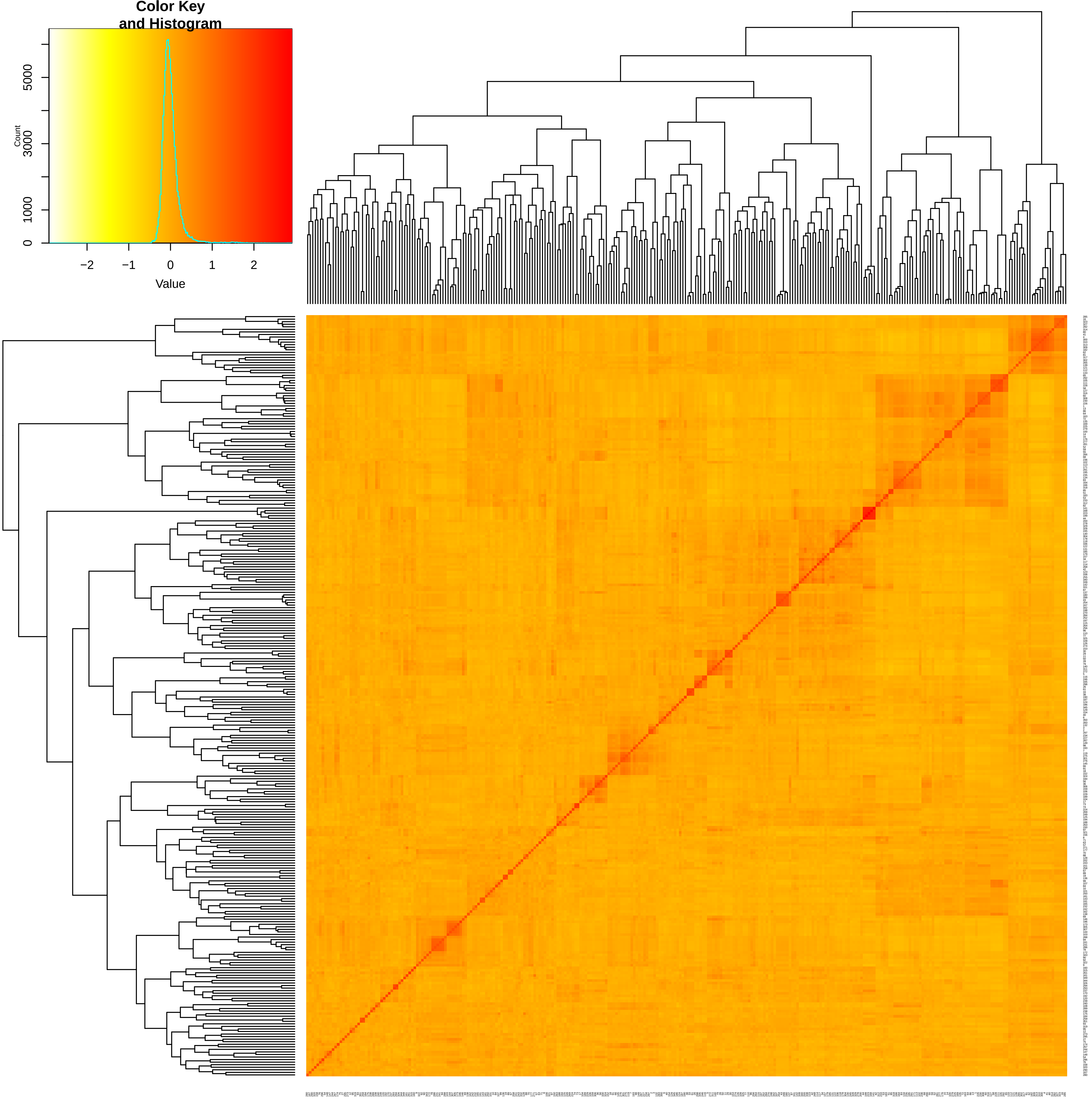
Color Key
and Histogram
Count
0
1000
3000
5000
−2
−1
0
1
2
Value
