# Supplementary figures and images for: Identification of Genomic Regions and Sources for Wheat Blast Resistance through GWAS in Indian Wheat Genotypes
Source: Genes (Basel). 2022 Mar 27;13(4):596. doi: 10.3390/genes13040596 (PMC9025667; doi:10.3390/genes13040596)

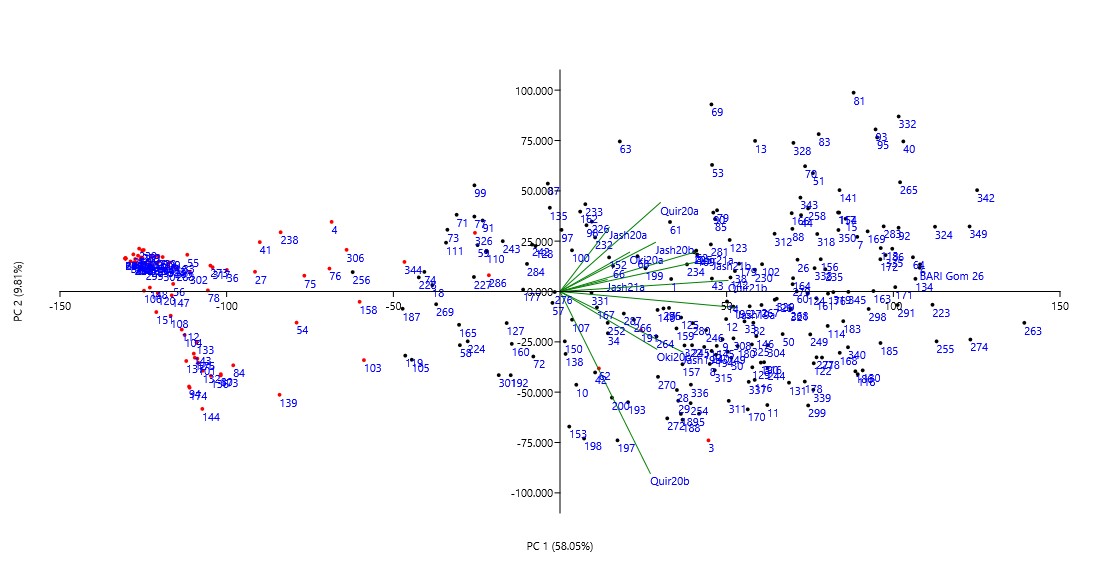

Supplement: Supplementary file 1 [file genes-13-00596-s001.zip › Figure S1. PCA with labels.jpg]

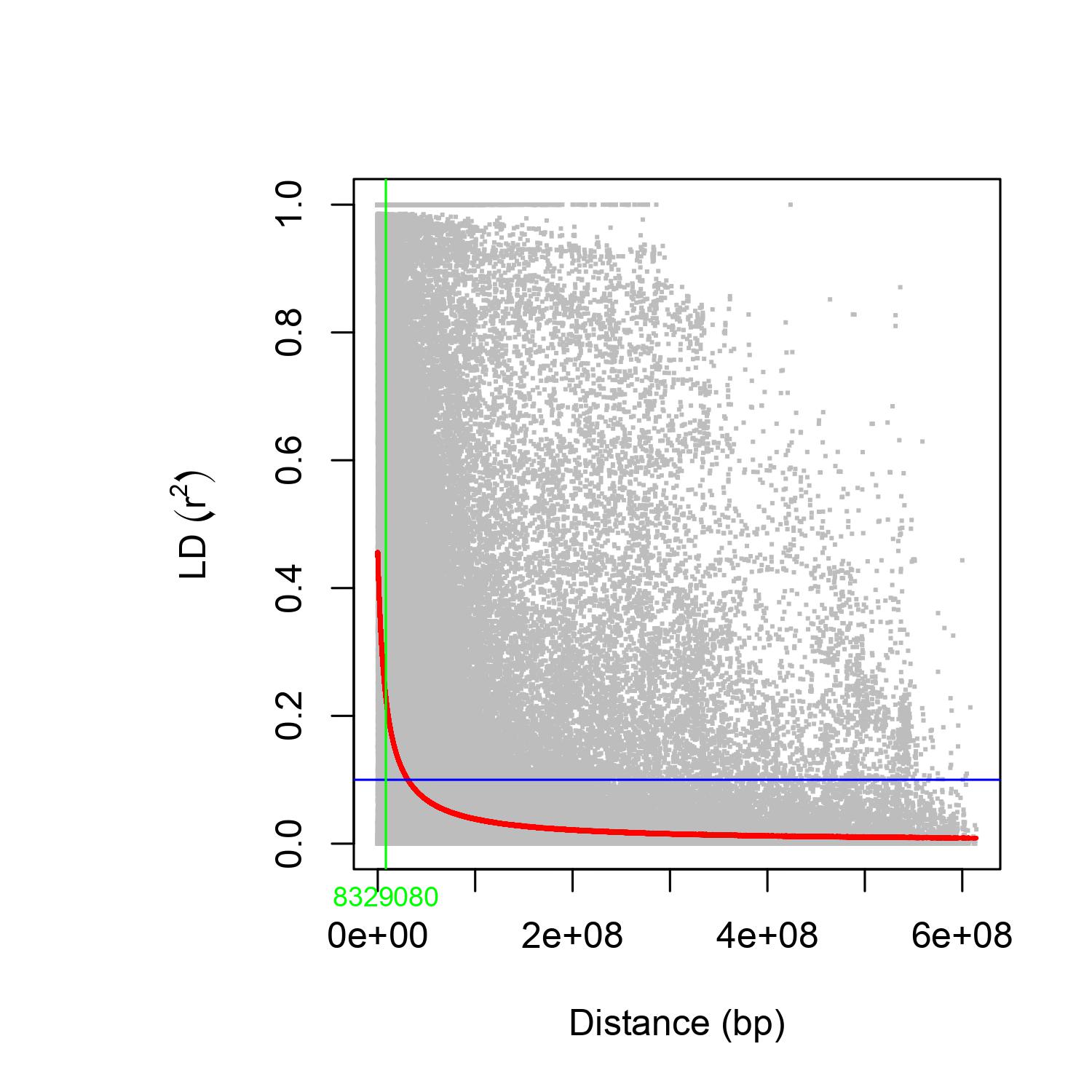

Supplement: Supplementary file 1 [file genes-13-00596-s001.zip › Figure S3. LD decay plot.jpg]

**HI 8819= HI 8713/ HI 8663**

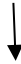

| S.No | Variety | Parentage         |
|------|---------|-------------------|
| 1    | HI 8713 | HD 4672 / PDW 233 |
| 2    | HI 8663 | HI 8177 / HI 8185 |

**HI 8713: HD 4672 / PDW 233**

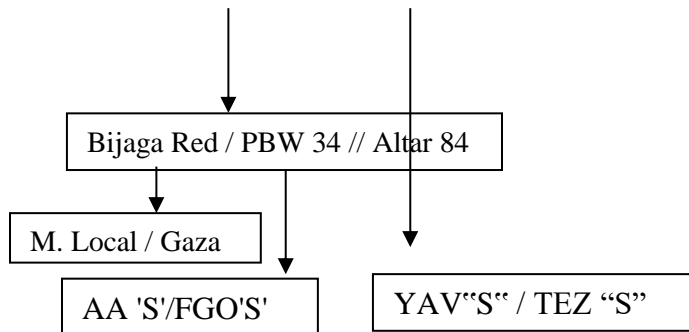

**HI 8663: HI 8177 / HI 8185**

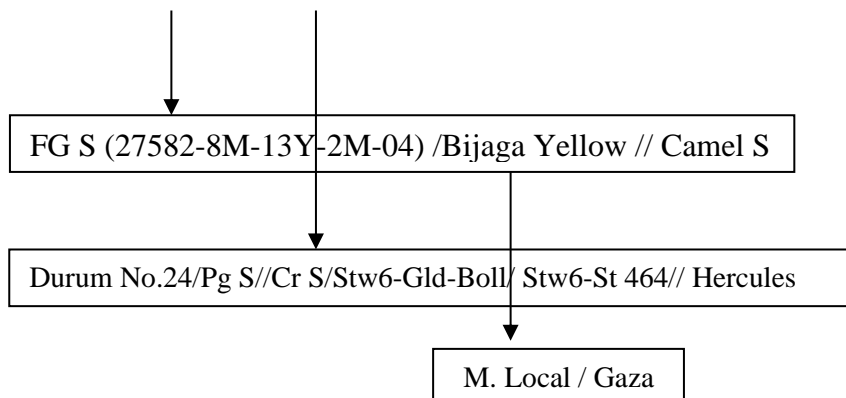

Supplement: Supplementary file 1 [file genes-13-00596-s001.zip › Figure S4. HI 8819 pedigree flowchart.pdf]
